# Complete mitochondrial genome of the freshwater monogonont rotifer *Brachionus rubens* (Rotifera, Brachionidae)

**DOI:** 10.1080/23802359.2019.1694853

**Published:** 2019-12-10

**Authors:** Beom-Soon Choi, Young Hwan Lee, Jin-Sol Lee, Erick O. Ogello, Hee-Jin Kim, Atsushi Hagiwara, Jae-Seong Lee

**Affiliations:** a Phyzen Genomics Institute, Seongnam, South Korea;; b Department of Biological Science, College of Science, Sungkyunkwan University, Suwon, South Korea;; c Department of Fisheries & Natural Resources, Maseno University, Kisumu, Kenya;; d Institute of Integrated Science and Technology, Nagasaki University, Nagasaki, Japan;; e Organization for Marine Science and Technology, Nagasaki University, Nagasaki, Japan

**Keywords:** Monogonont rotifer, complete mitochondrial genome, *Brachionus rubens*

## Abstract

The two complete mitochondrial genomes were sequenced from the freshwater monogonont rotifer *Brachionus rubens*. The genome sequences were 12,041 bp and 13,793 bp in size, and the gene order and contents were identical to those of the freshwater rotifer *B. rubens* China, but were different in three tRNA-Arg, tRNA-Ile, and tRNA-Leu between both *B. rubens* mitochondrial genomes, while *B. calyciflorus* had peculiar gene order in mitochondrial DNA I. Of 12 protein-coding genes (PCGs), one gene (*ND5*) had incomplete stop codons. Furthermore, the start codon of *ND4* and *CO2* gene was ATT, while the start codon of other PCGs was ATG. The base composition of 12 PCGs in *B. rubens* mitogenome showed 22.5% for A, 46.5% for T, 16.3% for C, and 14.7% for G, respectively.

To date, only a few complete mitochondrial genomes have been published in the freshwater rotifer *Brachionus* sp.; *Brachionus calyciflorus* (Nie et al. 2016) and *Brachionus rubens* China (GenBank KJ489417 and KJ489418), while several marine rotifer *Brachionus* sp. mitogenomes were reported (Suga et al. 2008 for *B. plicatilis*; Hwang et al. 2014 for *Brachionus koreanus*, and Kim et al. 2017 for *Brachionus rotundiformis*). *Brachionus rubens* is one of the major cosmopolitan freshwater rotifers and was considered as a model for environmental toxicology in response to toxic cyanobacterium (Geng et al. 2006; Geng and Xie 2008; Pérez-Morales et al. 2014), temperature and metal (Azuara-García et al. 2006; Montúfar-Meléndez et al. 2007), suggesting that this species can be an important member of freshwater zooplankton community as a sentinel species for ecotoxicology. The analysis of *B. rubens* mitochondrial genome is important to identify field-sampled and laboratory stocks. In this study, we identified two complete mitochondrial genomes of the monogonont rotifer *B. rubens* Japan to better understand the phylogenetic placement of the freshwater rotifer *Brachionus* clade.

The adult *B. rubens* were collected from the freshwater catchment area at Urakami, Nagasaki in Japan (32°79′90.72″N, 129°86′55.32″E) in August 2004 and maintained at the Laboratory of Professor Atsushi Hagiwara, Nagasaki University in Japan. The type was deposited in the ichthyological collection of the Faculty of Fisheries, Nagasaki University (FFNU) under the accession no. FFNU-Rot-0003. We sequenced 300 bp, 500 bp and 800 bp paired end library of *B. rubens* from whole body genomic DNA using the Illumina HiSeq 2500 platform (GenomeAnalyzer, Illumina, San Diego, CA). *De novo* assembly was conducted by SPAdes (version 3.13.0) (http://cab.spbu.ru/software/spades/) with K-mer auto. Of the assembled *B. rubens* 270,986 contigs with Newbler (version 2.9; identity 97) (http://www.454.com), seven mitochondrial contigs were obtained. After a manual curation of seven contigs with Consed (version 19.0) (http://www.phrap.org/consed/consed.html), two contigs were obtained to the mitochondrial DNA of *B. rubens*.

The complete mitochondrial genomes of *B. rubens* were 12,041 bp (mitochondrial DNA I; GenBank no. MN256531) and 13,793 bp (mitochondrial DNA II; GenBank no. MN256532) in size. The direction of 12 protein-coding genes (PGCs) of *B. rubens* Japan was identical to those of *B. rubens* China of the genus *Brachionus*, including the presence of nearly identical non-coding region (Identities: 5651/5731 = 98.25%) (Suga et al. 2008; Hwang et al. 2013). Of 12 PCGs, one gene (*ND5*) had incomplete stop codons. Furthermore, the start codon of *ND4* and *CO2* gene was ATT, while the start codon of other PCGs was ATG. The base composition of 12 PCGs in *B. rubens* Japan mitogenome showed 22.5% for A, 46.5% for T, 16.3% for C, and 14.7% for G, respectively. The mitochondrial genome A + T base composition (69.0%) of 12 PCGs was higher than G + C (31.0%), while the complete mitochondrial genome A + T base composition (67.5%) was higher than G + C (32.5%).

The placement of *B. rubens* Japan in the genus *Brachionus* with CO1 and Cytb was shown in Figure 1. *B. angularis* was clustered closely to *B. rubens* China. The gene order and contents of 12 PGCs were identical to those of the freshwater rotifer *B. rubens* China. However, interestingly, the order of tRNAs of three tRNA-Arg, tRNA-Ile, and tRNA-Leu was different between both *B. rubens* mitochondrial genomes, while the freshwater rotifer *B. calyciflorus* had peculiar gene order in mitochondrial DNA I. This indicates that the rearrangement of tRNAs is likely occurring in sporadic manner in the genus *Brachionus* (Hwang et al. 2014).

**Figure 1.**
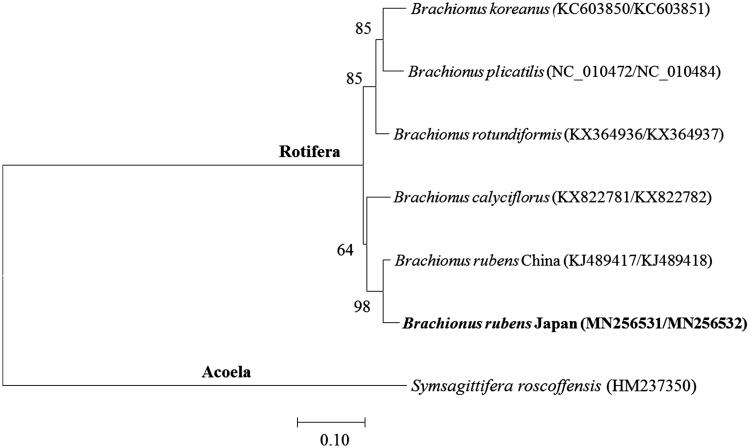
Phylogenetic analysis of the rotifer *Brachionus rubens* Japan mitochondrial DNA. We conducted a comparison of two mitochondrial DNA genes (CO1 and Cytb) of Acoela and Rotifera. Two mitochondrial DNA genes CO1 and Cytb were aligned by ClustalW. Maximum likelihood (ML) analysis was performed by Raxml 8.2.8 (http://sco.h-its.org/exelixis/software.html) with GTR + γ+I nucleotide substitution model. The rapid bootstrap analysis was conducted with 1,000 replications with 48 threads running in parallel. The Acoela served as an outgroup. Ln=-5316.105. Modified from Choi et al. (2019).
